# INTERDISCIPLINARY PUBLIC POLICY DISCUSSION SESSION

**DOI:** 10.1093/geroni/igac059.640

**Published:** 2022-12-20

**Authors:** George Taffet, Brian Lindberg

## Abstract

This interactive session is an interdisciplinary look at policy issues in aging with the speakers representing the four sections of GSA, ESPO, and AGHE. This session, organized by the GSA Public Policy Advisory Panel, will provide both GSA section leadership and attendees an opportunity to have an open dialogue on important public policy issues of significance in the field of aging.

